# A Multi-Parametric Islet Perifusion System within a Microfluidic Perifusion Device

**DOI:** 10.3791/1649

**Published:** 2010-01-26

**Authors:** Adeola F. Adewola, Yong Wang, Tricia Harvat, David T. Eddington, Dongyoung Lee, Jose Oberholzer

**Affiliations:** Department of Surgery,; Department of Bioengineering,

## Abstract

A microfluidic islet perifusion device was developed for the assessment of dynamic insulin secretion of multiple islets and simultaneous fluorescence imaging of calcium influx and mitochondrial potential changes. The device consists of three layers: first layer contains an array of microscale wells (500 μm diameter and 150 μm depth) that help to immobilize the islets while exposed to flow and maximize the exposed surface area of the islets; the second layer contains a circular perifusion chamber (3 mm deep, 7 mm diameter); and the third layer contains an inlet-mixing channel that fans out before injection into the perifusion chamber (2 mm in width, 19 mm in length, and 500 μm in height) for optimizing the mixing efficiency prior to entering the perifusion chamber. The creation of various glucose gradients including a linear, bell shape, and square shapes also can be created in the microfluidic perifusion network and is demonstrated.

**Figure:**
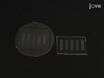


## Protocol

### A. Microfabrication of 3-layer microfluidic perifusion device

#### Bottom Well Master Protocol (150 μm deep wells)

Clean the wafer using a razor blade if needed. Clean with Acetone, Methanol, and IPA. Perform plasma treatment at 50 Watts for 30 s.
Spin SU8-100 @ 2000 rpm. [Step 1: 500 rpm, 10 s, 100 rpm/s, Step 2:  2000 rpm, 30 s, 300 rpm/s].[NOTE: do not hold the wafer with tweezers after spinning SU8]
Soft bake the wafer at 65 °C for 20 min and at 95 °C for 50 min.
The wafer is exposed to UV light through a desired mask. Dose for 150 μm height is 650 mJ/cm^2^.
Post exposure bake the wafer at 65 °C for 1 min and at 95 °C for 12 min.
Develop the wafer in SU8 developer for 15 min.

#### Microchannel Master Protocol (500 μm deep wells)

Clean the wafer using a razor blade if needed. Clean with Acetone, Methanol, and IPA. Perform plasma treatment at 50 Watts for 30 s.
Spin SU8-2150 @ 1000 rpm. [Step 1: 500 rpm, 10 s, 100 rpm/s, Step 2:  1000 rpm, 30 s, 300 rpm/s].
Soft bake the wafer at 65 °C for 15 min and at 95 °C for 2 hrs and 30 min.
The wafer is exposed to UV light through a desired mask. Exposure dose for 650 μm height is 685 mJ/cm^2^.
Post exposure bake the wafer at 65 °C for 5 min and at 95 °C for 35 min.
Develop the wafer in SU8 developer for 20-30 min.

#### PDMS solution preparation

Polydimethylsiloxane (PDMS) solution is prepared by thoroughly mixing 10 parts of silicone elastomer with 1 part of curing agent of a standard Sylgard 184 kit.
The bubbles generated in the PDMS solution during the mixing process are removed using a vacuum desiccator.
The bubble-free PDMS solution is slowly dispensed onto the SU8 masters and an empty Petri dish for the third layer.
The temperature of the hot plate is set to 75 °C and the PDMS is cured at this temperature for 2 hrs
Inlets, outlets and exchange wells are punched out using the appropriate size hole puncher.
The layers are bonded together on a glass slide (size 0.1 mm) using a handheld plasma device.

### B. Experimental setup

Flow 70 % ethanol through the micro-device to sterilize. Flow DI water to wash out the ethanol. Perfuse 50 ml of 0.5 %BSA through the device to prevent non specific adsorption of insulin to the microchannel walls.
The glucose ramp gradient and other related gradients generated by LabView software that communicates with the syringe pumps are tested to make sure the gradients are stable.
25-30 mice islets were incubated with 5 μM Fura-2/AM (a calcium indicator, Molecular probes, CA) and 2.5 μM Rhodamine 123 (Rh123, a mitochondrial potentials indicator, Sigma, MO) for 30 min at 37 °C in Krebs-Ringer buffer (KRB) containing 2 mM glucose
The mice islets are carefully pipetted into microfluidic perifusion device through the inlet port.
The microfluidic network is then setup by connecting the inlet to the syringe pumps using Tygon tubing and a Y-connector and the outlet to fraction collector. The device sits on a heating stage (37 °C) on the microscope and the inlet tubing is heated on a hotplate to keep the temperature of the chamber and solution at 37 °C.
Immediately after setup, the mice islets are perifused with KRB containing 2 mM glucose for 10 min and then a glucose ramp (2mM   25mM) for 25 min.
Time-lapse images are collected and analyzed every 15 s by SimplePCI software. The perifusate is also collected every minute using a fraction collector to analyze the insulin secretion using ELISA kit.

### Representative Results

Mouse islets were perifused with a linear gradient of 2-25 mM glucose.  As shown in Figure 1, calcium influx and insulin secretion are triggered after about 13 minutes of perifusion, corresponding to 6 mM glucose.  Changes in Mitochondrial potentials are seen earlier as expected, at about 11 minutes.  This data demonstrates the advantage of using this microfluidic network to characterize islet physiology.


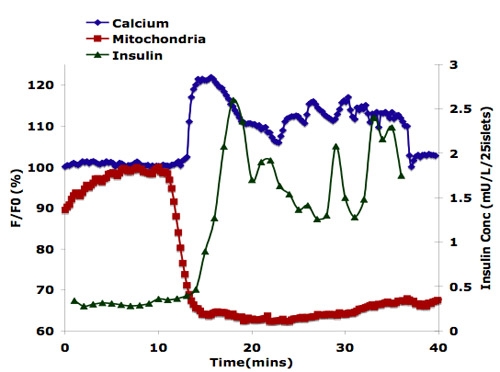
 **Figure 1.** Physiological responses to islet cell stimulation.

## Discussion

Traditional islet perifusion systems (macroscale and microscale) have some limitations including complex of setup and design, high technical requirements, and difficulty to create user-prescribed chemical gradients in the system. The microfluidic perifusion system described here overcomes these limitations with simple geometry of design and fabrication. More important, this system can be integrated with fluorescence imaging approach that provide as a unique tool to study islet physiology. The system demonstrated with high signal-noise ratio and spatial-temporal resolution of these fluorescence signals. The current prototype device also can hold multiple perifusion setups in one chip and provide different types of microenvironments for widespread application purposes.
